# The preferred conformation of *erythro-* and *threo-*1,2-difluorocyclododecanes

**DOI:** 10.3762/bjoc.8.143

**Published:** 2012-08-10

**Authors:** Yi Wang, Peer Kirsch, Tomas Lebl, Alexandra M Z Slawin, David O'Hagan

**Affiliations:** 1 EastChem School of Chemistry, University of St Andrews, St Andrews, KY16 9ST, UK; 2 Merck KGaA, Frankfurter Str. 250, 64293 Darmstadt, Germany

**Keywords:** alicyclic chemistry, conformational analysis, cyclododecane, ^19^F NMR, organo-fluorine chemistry, transannular interactions

## Abstract

Cyclododecane adopts a square-like structure with corner and edge CH_2_ groups. In this study e*rythro-* and *threo*-1,2-difluorocyclododecanes were prepared to explore whether the two vicinal C–F bonds, with different relative configurations, preferably locate at corner/edge or edge/edge locations. Conformational analysis comparing the diastereoisomers was explored by using a combination of ^19^F{^1^H} NMR spectroscopy, computational studies and, in the case of the *threo* isomer, X-ray structural analysis. In the lowest energy conformers for both diastereoisomers the vicinal C–F bonds are located corner/edge, rather than edge/edge. These structures avoid placing a C–F bond *endo* into the ring, and appear to benefit from C–CHF–C angle widening, which relaxes 1,4-H,H transannular interactions.

## Introduction

**Figure 1:**
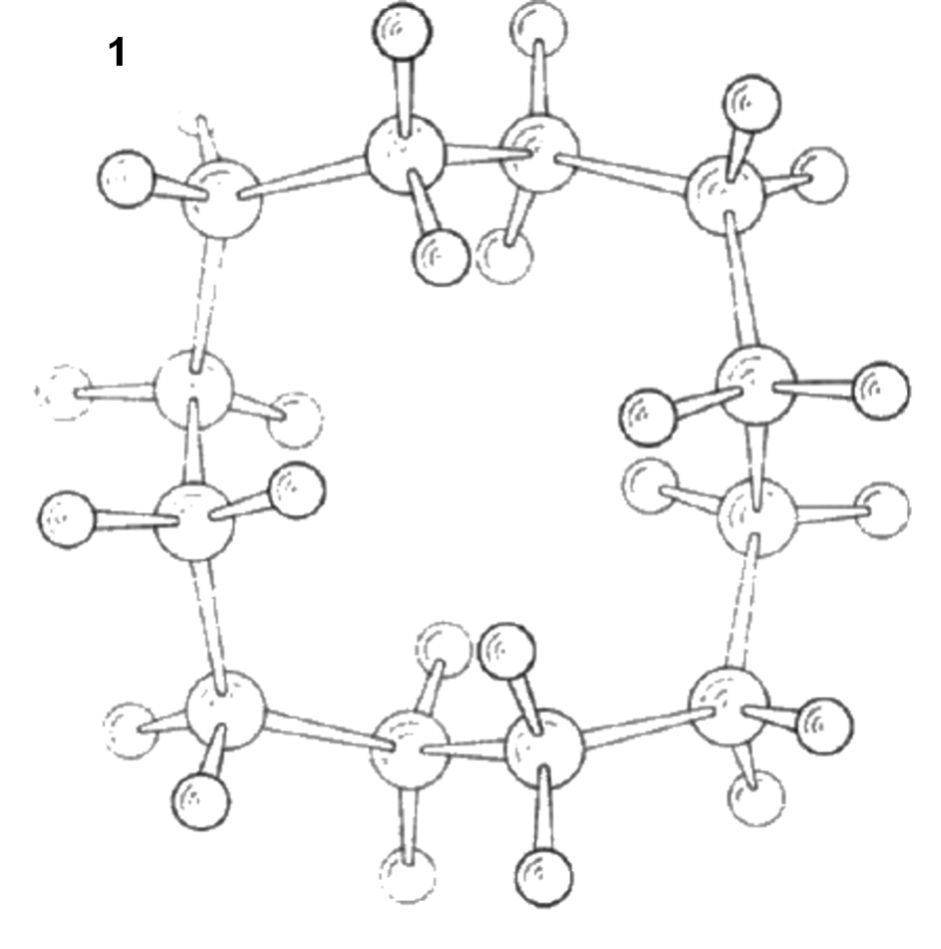
The Dunitz and Shearer structure of cyclododecane (**1**) [1–2]. There are four *endo* hydrogens above and four below the plane of the ring, which are tensioned as they are within van der Waals contact distances. Thus the ring adopts a [3333] square-type structure. (Reproduced with permission from [1]; Copyright © 1960 Verlag GmbH & Co KGaA, Weinheim).

The conformation of cyclododecane (**1**) in the solid state was first reported by Dunitz and Shearer in 1960 [1–2]. They showed that cyclododecane has a square topology, which can be classified as a [3333] type structure [3–4]. Their conclusion was derived from X-ray diffraction data, which could not fully resolve the structure due to a high level of disorder, but the diffraction data was used as the basis of a further computational analysis, and the structure in Figure 1 emerged as their consensus structure [5–6]. The structure is tensioned by transannular interactions in which there are four *endo* hydrogens, one on each edge pointing into the ring on the top face, and four more *endo* hydrogens pointing into the ring on the lower face. Each set of hydrogens forms a square, and they are spaced at van der Waals contact distances (~2.1–2.2 Å) to their contacted neighbours, situated at 1,4-positions on adjacent edges of the ring. This structure has two distinct types of methylene group, those located at corners of the ring and those at edges.

**Figure 2:**
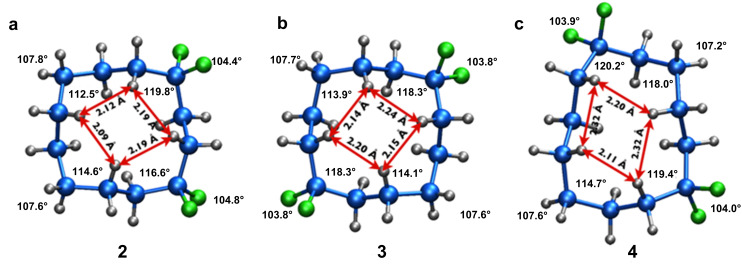
Crystal structures of (a) 1,1,4,4- (b) 1,1,7,7- and (c) 1,1,6,6-tetrafluorocyclododecanes (**2**–**4**) , illustrating the corner preference of the CF_2_ groups. Structures **2** and **3** adopt a [3333] conformation, whereas **4** adopts a distorted [4332] conformation. Fluorine atoms are presented in green [7].

Recently we prepared and explored the conformation of cyclododecane ring systems carrying either one or two difluoromethylene (CF_2_) groups in place of methylenes (CH_2_) [7]. The study revealed that the CF_2_ groups always occupied corner positions. This was deduced by a combination of ^19^F NMR, X-ray structure analysis and theory studies. Two reasons emerged for this. Firstly, if a C–F bond did project into the ring (*endo*), then the increased size of the fluorine atom relative to hydrogen increases the transannular 1,4-H,F relative to 1,4-H,H strain, by placing a fluorine in the square of hydrogen atoms on the top (or bottom) face of the ring. Secondly, the electronegativity of the fluorines within the CF_2_ group has the effect of distorting the sp^3^ geometry and widening the tetrahedral C–CF_2_–C angle from around 113° to about 116–118° [7–9]. This angle widening has the effect of lengthening/relaxing the 1,4-H,H transannular contacts between the transverse *endo* hydrogens, thus leading to a lower-energy ring structure. As a consequence of these two effects, the preference for a corner over an edge location is very strong, and thus when the CF_2_ groups are spaced 1,4 to each other or 1,7 to each other within the ring, they occupy adjacent and opposite corners of the ring, respectively, and form stable ring systems. This is illustrated in the X-ray structures in Figure 2. However when the CF_2_ groups were placed 1,6 to each other, a deliberate mismatch with respect to corner locations, the ring structure then became a distorted [4332] ring system, which is a structure that is more achievable than placing a CF_2_ group at an edge position of a [3333] ring system.

**Figure 3:**
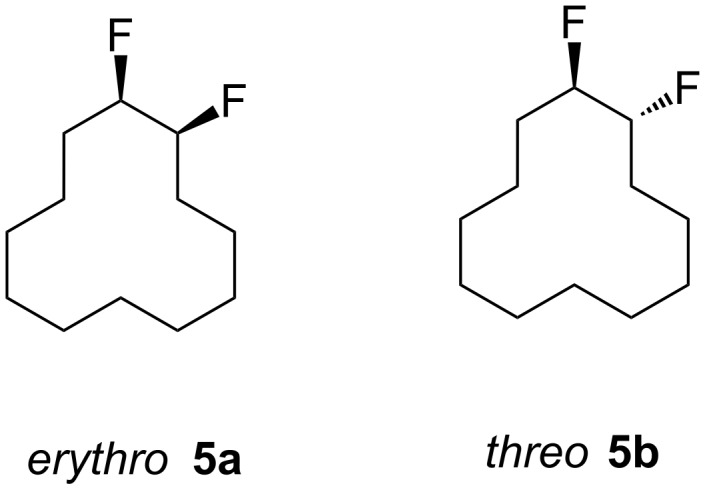
*Erythro-* and *threo*-1,2-difluorocyclododecanes (**5a** and **5b**).

In this study we separated the geminal fluorine atom of the CF_2_ group to generate vicinal fluorines in order to explore the conformational preference of the *erythro-* and *threo*- diastereoisomers of 1,2-difluorocyclododecanes **5a** and **5b** (Figure 3).

In the case of *erythro-* and *threo*-1,2-difluorocyclododecanes (**5a** and **5b**, respectively) it may be expected that the *threo* isomer **5b** will adopt a conformation whereby the vicinal fluorines occupy a central edge/edge location, such that both C–F bonds project *exo* to the ring, and do not increase the torsional strain. Also for the *threo* isomer **5b**, the C–F bonds may benefit from hyperconjugative interactions with the anti*-*periplanar C–H bonds, similar to that found in 1,2-difluoroethane in the well know *gauche* effect [10–11]. On the other hand, the *erythro* stereoisomer **5a** would have a C–F bond projecting into the ring in an *endo* manner, if the vicinal fluorines were edge/edge located, and thus it is anticipated that the *erythro* isomer **5a** will adopt a corner/edge location for the C–F bonds rather than an edge/edge location.

In order to test these hypotheses it was necessary to prepare different the diastereoisomers, *erythro*- and *threo*-1,2-difluorocyclododecane, and then subject them to conformational analysis by ^19^F NMR and X-ray structural analyses. A computational study was also carried out to explore the relative energies of the candidate edge/edge and edge/corner conformers.

## Results and Discussion

### Synthesis

**Scheme 1:**
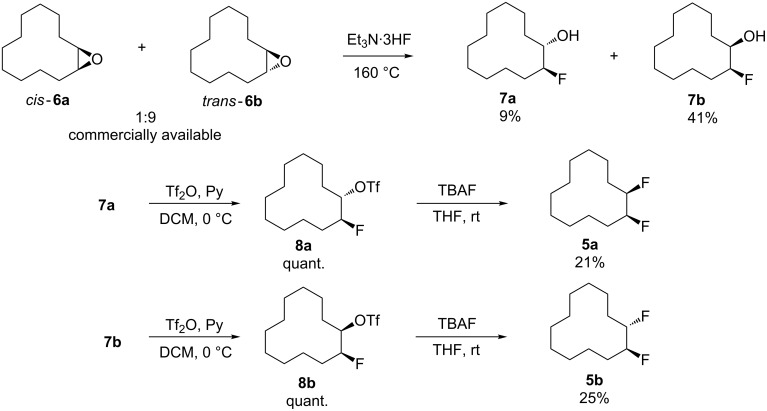
Synthetic routes to *erythro-* (**5a**) and *threo*-1,2-difluorocyclododecane (**5b**).

**Figure 4:**
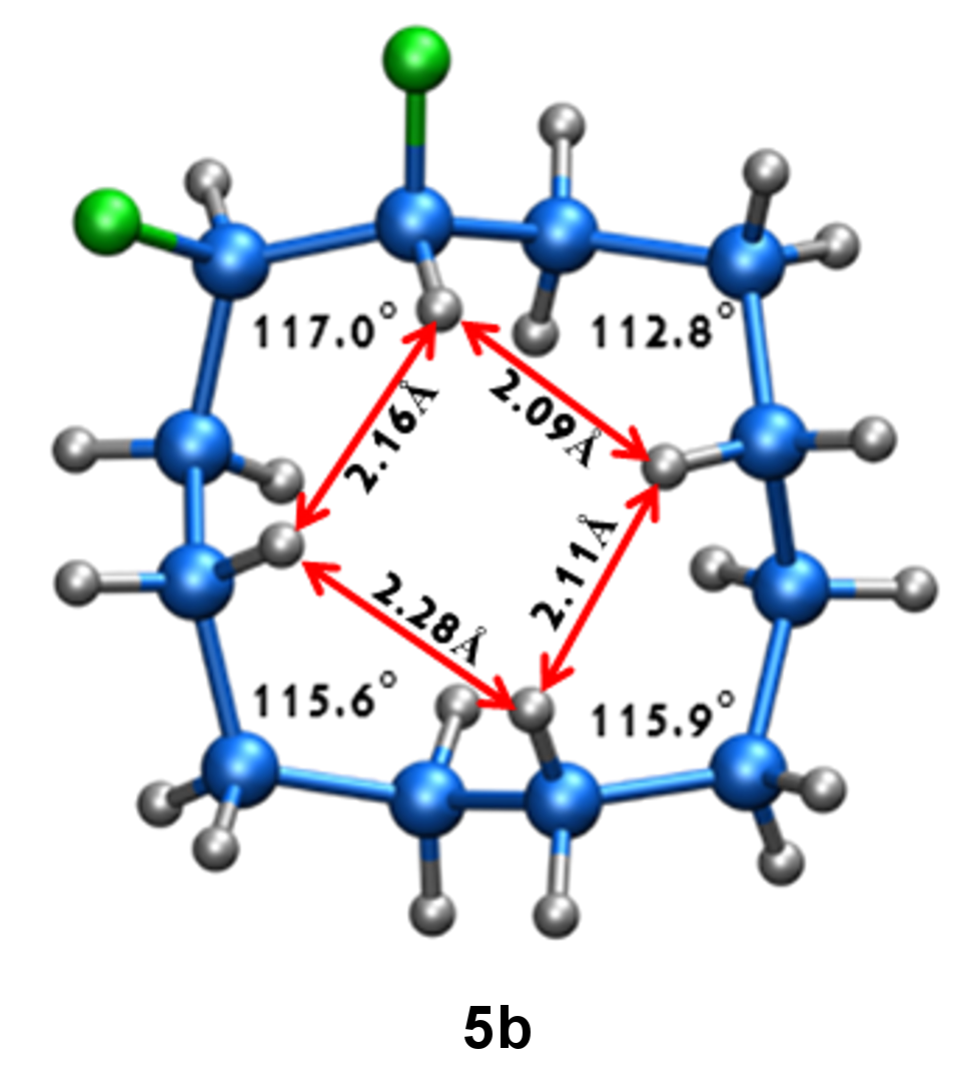
X-ray crystal structure of *threo*-1,2-difluorocyclododecane (**5b**) showing corner angles and representative transannular contact distances.

The synthetic route to *erythro-* (**5a**) and *threo*-1,2-difluorocyclododecanes (**5b**) is shown in Scheme 1. A (1:9) mixture of *cis-* and *trans*-epoxides was treated with triethylamine trihydrofluoride [12]. This afforded diastereoisomeric fluorohydrins **7a** and **7b**, which could be readily separated by chromatography. Each fluorohydrin was then treated with triflic anhydride [13–14], to generate the corresponding triflates **8a** and **8b**. Treatment of **8a** and **8b** with tetrabutylammonium fluoride (TBAF) in THF was stereospecific and independently generated the *erythro-* or *threo*-1,2-difluorocyclododecanes **5a** and **5b**. These compounds were white solids. In the case of the *threo* isomer only, a suitable crystal was grown such that an X-ray structure could be solved. The resultant structure, which confirmed the *threo* stereochemistry, is shown in Figure 4. Notably one of the C–F bonds occupies a corner location, inconsistent with our preconceived expectation of a edge/edge conformation. This corner/edge conformation appears to be favoured in the solid state over the edge/edge conformation. Also the C–CHF–C angle of 117.0° indicates a small rehybridisation tendency towards a wider angle, observed more dramatically with the CF_2_ group [7–9], and with concomitant release of angle strain.

### Variable-temperature (VT) ^19^F NMR

**Figure 5:**
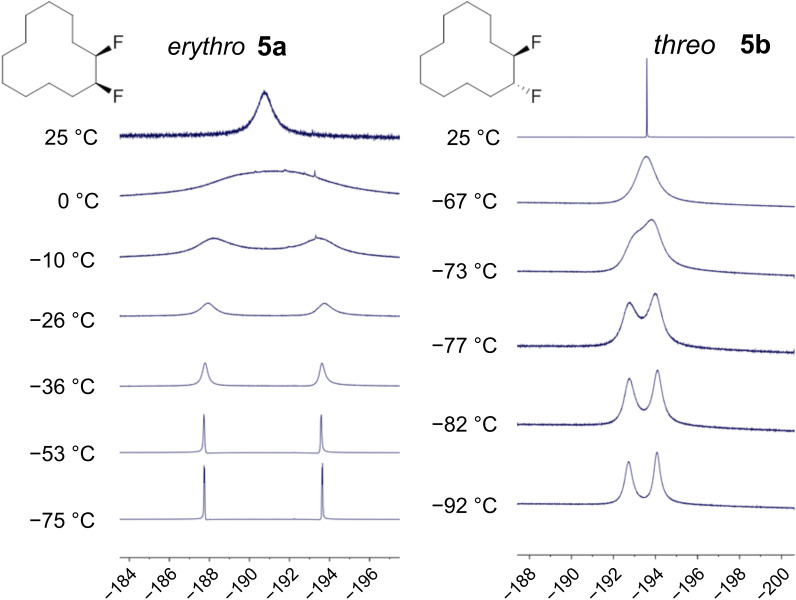
Variable-temperature ^19^F{^1^H} NMR of *erythro-* (**5a**) and *threo*-1,2-difluorocyclododecane (**5b**).

Low-temperature (CD_2_Cl_2_, 180 K) ^19^F{^1^H} NMR experiments were carried out on both the *erythro* and *threo* isomers. The spectra are shown in Figure 5. In each case at room temperature there is a single fluorine resonance; however, on lowering of the temperature the fluorine signal resolves into an AB-system, indicating nonequivalent fluorine environments and, thus, corner/edge locations of the C–F bonds in each case. Vicinal edge/edge conformations would result in the magnetic equivalence of the fluorine atoms, but this is not observed. Both isomers **5a** and **5b** display a single resonance at room temperature (25 °C), indicating rapid ring interconversion on the NMR timescale. Rate constants for the ring interconversions were determined by complete lineshape analysis of the ^19^F NMR spectra recorded across the temperature range 180–295 K.

Fitting the experimental data to the Eyring equation [15] allowed determination of the activation parameters (see Table 1 and Supporting Information File 1).

**Table 1:** The activation parameters of *erythro-* (**5a**) and *threo-* (**5b**) 1,2-difluorocyclododecanes.

isomer	Δ*G*^#^ kcal·mol^−1^	Δ*H*^#^ kcal·mol^−1^	Δ*S*^#^ kcal·mol^−1^K^−1^

*erythro* **5a**	10.5 ± 1.5	13.5 ± 0.6	10.0 ± 2.7
*threo ***5b**	9.47 ± 1.1	6.57 ± 0.3	−9.73 ± 2.9

The overall free energy change (Δ*G*^#^) is similar in each case and both the *erythro* **5a** and *threo* **5b** stereoisomers have conformational energy barriers ~2–3 kcal·mol^−1^ higher than cyclododecane itself (7.3 kcal·mol^−1^) indicating that fluorine introduces some conformational stability. The enthalpy difference (Δ*H*^#^) is significant between the isomers. The theory calculations described below suggest that the *erythro* isomer **5a** is more stable than the *threo* isomer **5b** in the ground state, thus this is most probably the major contributor to the enthalpy difference. The opposite sign in entropy (Δ*S*^#^) for each isomer makes a relatively small contribution to the overall free energy; however, the positive value for the *erythro* isomer is perhaps unexpected for progression towards a transition state. This may arise as a result of desolvation for this isomer.

### Computational study

**Figure 6:**
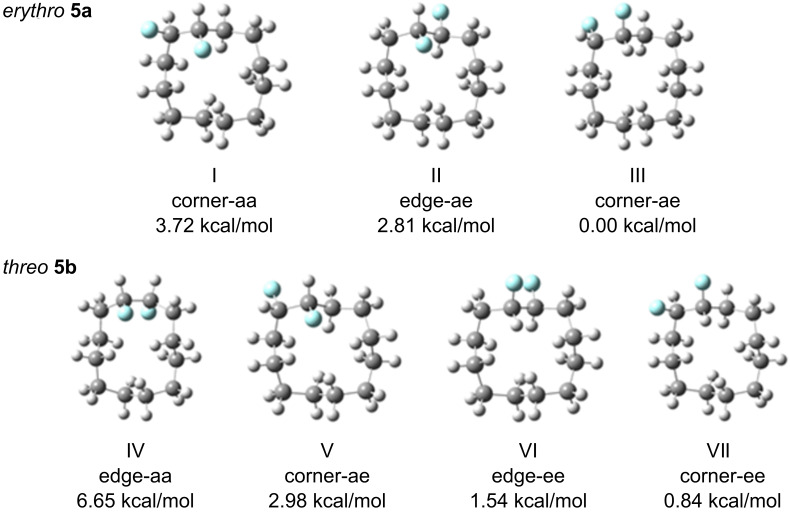
Calculated relative energies of the conformations of the *erythro* (**5a**) and *threo* (**5b**) stereoisomers of 1,2-difluorocyclododecane.

In order to explore conformer energies further, a theoretical study MP2/6-311+G(2d,p)//B3LYP/6-311+G(2d,p)+ZPE) [16] was carried out to assess relative ground-state energies of candidate conformers. The structures and relative energies for the *erythro* **5a** and *threo* **5b** isomers are shown in Figure 6. These data indicate that the corner/edge conformers are more stable than the alternative edge/edge conformers for each stereoisomer. This is consistent with the conclusions from the experimental VT ^19^F NMR study.

For the *erythro* stereoisomer **5a**, three conformers I–III were considered. Conformers I and II each have a fluorine pointing into the ring (*endo*), and thus there is an increase in transannular ring strain, raising the energy of these conformers by 2.81 and 3.72 kcal·mol^−1^ respectively above conformer III, the lowest in energy. For the *threo* stereoisomer **5b**, four conformers, IV–VII were considered. Conformers IV and V, which have two and one *endo* fluorine, respectively, are highest in energy. In particular, conformer IV with two *endo* fluorines has a ground-state energy of 6.65 kcal·mol^−1^, the highest of all of those examined, illustrating the additive and negative impact of placing fluorines into *endo* orientations. Conformers VI and VII are lower in energy. It was anticipated at the outset that conformer VI may be the most favoured for the *threo* isomer; however, this does not appear to be the case, although the energy difference between the lowest-energy corner/edge conformer VII and edge/edge conformer VI is relatively small at VI − VII = 0.7 kcal·mol^−1^. This theoretical observation is supported by the VT ^19^F{^1^H} NMR study, which indicates nonequivalent fluorines consistent with a corner location. Also the structure of conformer VII is almost identical to that obtained experimentally by X-ray structure analysis (Figure 4).

It is not immediately obvious why *threo* **5b** conformer VII is favoured (lower in energy) over conformer VI, although the energy difference is small (~0.7 kcal·mol^−1^). It is noteworthy that the fluorine atoms are a little closer in intramolecular distance in VII (F···F 2.68Å) compared to VI (F···F 2.75Å), and thus electrostatic repulsion does not appear to be the discriminating factor. One origin for this preference, which emerges from the theoretical study, may be the widening of the C−CHF−C angle (115.62°) in VII. The introduction of fluorine alters the hybridisation at carbon [5], and this should relieve angular strain in these tensioned ring systems relative to a strained C−CH_2_−C angle of ~115° (see Table 2).

**Table 2:** The corner C–C–C angles for conformers VI and VII of the *threo* isomer **5b**. The C–CHF–C angle (115.62°) is the largest; however some C–CH_2_–C angles are clearly strained at ~115.0°.

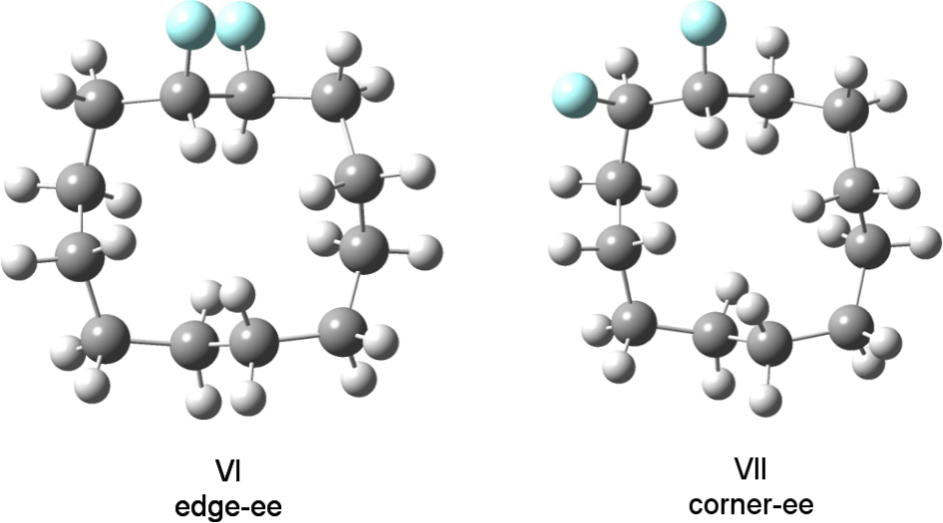				

*threo ***5b**	top left corner [°]	top right corner [°]	bottom left corner [°]	bottom right corner [°]

VI	113.42 (CH_2_)	113.41 (CH_2_)	115.16 (CH_2_)	115.21 (CH_2_)
VII	115.62 (CHF)	113.91 (CH_2_)	115.09 (CH_2_)	114.32 (CH_2_)

## Conclusion

The *erythro* (**5a**) and *threo* (**5b**) isomers of 1,2-difluorocyclododecane were synthesised, and their preferred conformers were explored experimentally by ^19^F NMR, and X-ray structure analysis. A computational study was also carried out to establish favoured conformations and their relative ground-state energies. A particular focus of the study examined whether the vicinal C–F bonds prefer to adopt corner/edge or edge/edge locations of the [3333] ring system. For each diastereoisomer it emerged that one of the C–F bonds adopts a corner location. The second orientates *exo* to the ring. When the C–F bond projects *endo* into the ring, the energy of the system is raised by between 2.0–3.0 kcal·mol^−1^ and is disfavoured. In the case of the *threo* isomer **5b**, the outcome was less easy to predict, as an edge/edge structure can be achieved with both C–F bonds *exo* to the ring, and with each C–F bond benefiting from antiperiplanar C–H/C–F hyperconjugative interactions. However it would appear that the corner/edge structure is still favoured for the *threo* isomer **5b**, as the C–C–C bond angles of ~115°, which occur in these tensioned ring systems, are inherently less strained, due to rehybridisation/angle widening, if they carry a central fluorine atom.

## Experimental

Preparation of **7a** and **7b**: Commercially available 1,2-epoxycyclododecane (**6**) (5 mmol, 0.91 g, 9:1 *trans*/*cis*) and Et_3_N·3HF (4.0 g, 25 mmol) was added to a Teflon-coated reactor and stirred at 160 °C for 24 h. After cooling down, the reaction mixture was quenched with sat. NaHCO_3_ solution (50 mL) and extracted into diethyl ether (3 × 20 mL). The organic layers were combined and dried (MgSO_4_), and then concentrated under vacuum. Purification over silica gel, eluting with hexane and diethyl ether (90:10), yielded *trans*-2-fluorocyclododecanol (**7a**) (91 mg, 9%) and *cis*-2-fluorocyclododecanol (**7b**) (420 mg, 41%) as a white crystalline solid.


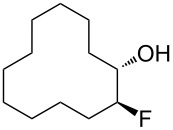


**7a**: mp 64–65 °C; ^1^H NMR (400 MHz, CDCl_3_) δ_H_ 4.56 (ddt, *J =* 49.4, 8.7, 4.2 Hz, C*H*F), 3.94–3.86 (m, 1H, CHO*H*), 2.27 (t, *J =* 3.5 Hz, 1H, C*H*OH) and 1.92–0.84 (m, 20H, 10 × CH_2_); ^13^C NMR (100 MHz, CDCl_3_) δ_C_ 95.3 (d, *J =* 166 Hz, CHF), 71.4 (d, *J =* 18 Hz, CHOH), 28.7 (d, *J =* 5.5 Hz, CH_2_), 27.8 (d, *J =* 21 Hz, CH_2_), 24.0 (d, *J =* 1.6 Hz, 2 × CH_2_), 23.7 (d, *J =* 2.8 Hz, 2 × CH_2_), 22.6 (d, *J =* 2.9 Hz, 2 × CH_2_), 20.6 (CH_2_), 20.4 (d, *J =* 2.8 Hz, CH_2_); {^1^H}^19^F NMR (376 MHz, CDCl_3_) δ_F_ −194.0 (CHF); LRMS–ESI (*m*/*z*): [M + Na]^+^ calcd for C_12_H_23_OFNa, 225.16; found, 225.06.


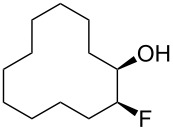


**7b**: mp 84–86 °C; ^1^H NMR (400 MHz, CDCl_3_) δ_H_ 4.70 (dtd, *J =* 47.3, 6.0, 1.7 Hz, 1H, C*H*F), 3.99–3.88 (m, 1H, CHO*H*), 1.81 (d, *J =* 5.4 Hz, 1H, C*H*OH) and 1.83–1.33 (m, 20H, 10 × CH_2_); ^13^C NMR (75 MHz, CDCl_3_) δ_C_ 95.4 (d, *J =* 168 Hz, CHF), 71.6 (d, *J =* 20 Hz, CHOH), 28.3 (d, *J =* 7 Hz, CH_2_), 25.2 (d, *J =* 21 Hz, CH_2_), 24.6 (CH_2_), 24.3 (CH_2_), 23.8 (CH_2_), 23.6 (CH_2_), 21.7 (2 × CH_2_), 21.4 (d, *J =* 5 Hz, CH_2_), 21.3 (CH_2_); {^1^H}^19^F NMR (376 MHz, CDCl_3_) δ_F_ −191.1 (CHF); LRMS–ESI (*m*/*z*): [M + Na]^+^ calcd for C_12_H_23_OFNa, 225.16; found, 225.07.


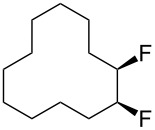


*Erythro* isomer **5a**: To a solution of **7a** (0.11 g, 0.5 mmol) in DCM (5 mL) at 0 °C was added pyridine (80 µL, 1 mmol, 2 equiv) and Tf_2_O (130 µL, 0.7 mmol, 1.4 equiv). The resulting mixture was stirred for 1 h and the solvent was removed under vacuum. The residue containing the *trans-*fluorotriflate **8a** was dissolved in THF (3 mL), and TBAF solution (1 mL, 1 M in THF, 1 mmol) was added dropwise. The reaction mixture was stirred at rt for 48 h and monitored by ^19^F NMR. Purification over silica gel, eluting with 1% diethyl ether in cyclohexane yielded *erythro*-difluorocyclododecane **5a** (21 mg, 21%) as a white solid: mp 57 °C; ^1^H NMR (400 MHz, CDCl_3_) δ_H_ 4.81–4.64 (tdd, *J =* 48.3, 24.1, 6.3 Hz, 2H, 2 × C*H*F), 1.80–1.73 (m, 4H, 2 × CH_2_), 1.44–1.33 (m, 16H, 8 × CH_2_); ^13^C NMR (75 MHz, CDCl_3_) δ_C_ 92.5 (dd, *J* = 174, 20 Hz, 2 × CHF), 26.9 (2 × CH_2_), 25.6 (dd, *J =* 21, 6 Hz, CH_2_), 24.0 (4 × CH_2_), 21.6 (2 × CH_2_), 21.0 (CH_2_), 20.9 (CH_2_); {^1^H}^19^F NMR (376 MHz, CDCl_3_) δ_F_ −191.0 (CF); HRMS–ESI (*m*/*z*): [M + Na]^+^ exact mass calcd for C_12_H_22_F_2_Na, 227.1587; found, 227.1591.


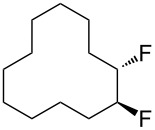


*Threo* isomer **5b**: Similar to the *erythro* compound, **5b** was obtained from *cis-***7b** in 25% as a white solid: mp 61 °C; ^1^H NMR (400 MHz, CDCl_3_) δ_H_ 4.81–4.64 (m, 2H, 2 × C*H*F), 2.10–1.23 (m, 20H, 10 × CH_2_); ^13^C NMR (100 MHz, CD_2_Cl_2_) δ_C_ 92.4 (dd, *J* = 176, 19 Hz, 2 × CHF), 29.7 (CH_2_), 27.8 (dd, *J =* 15, 12 Hz, 2 × CH_2_), 24.0 (2 × CH_2_), 23.7 (2 × CH_2_), 22.5 (2 × CH_2_), 20.4 (CH_2_); {^1^H}^19^F NMR (376 MHz, CDCl_3_) δ_F_ −193.6 (CF); HRMS–ESI (*m*/*z*): [M + Na]^+^ exact mass calcd for C_12_H_22_F_2_Na, 227.1587; found, 227.1595.

## Supporting Information

The Supporting Information contains NMR spectra and results of the differential scanning calorimetry, variable-temperature NMR, and computational studies.

File 1 Additional data.
