# Supplementary material for: The preferred conformation of erythro- and threo-1,2-difluorocyclododecanes
Source: Beilstein J Org Chem. 2012 Aug 10;8:1271–8. doi: 10.3762/bjoc.8.143 (PMC3458748; doi:10.3762/bjoc.8.143)
Supplement: File 1 — Additional data. [file Beilstein_J_Org_Chem-08-1271-s001.pdf]

Supporting Information

for

The preferred conformation of *erythro*- and *threo*-1,2-difluorocyclododecanes

Yi Wang[1], Peer Kirsch[2], Tomas Lebl[1], Alexandra M. Z. Slawin[1] and David O'Hagan*[1]

[1]School of Chemistry, University of St Andrews, St Andrews, KY16 9ST, UK and [2]Merck KGaA, Frankfurter Str. 250, 64293 Darmstadt, Germany.

Email: David O'Hagan - do1@st-andrews.ac.uk

*Corresponding author

Additional data

Section 1 NMR spectra……S2

Section 2 Differential scanning calorimetry ……S6

Section 3 Variable-temperature NMR……S7

Section 4 Computational studies……S9

Section 1 NMR spectra

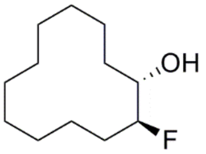

trans-**7a**

^{1}H NMR

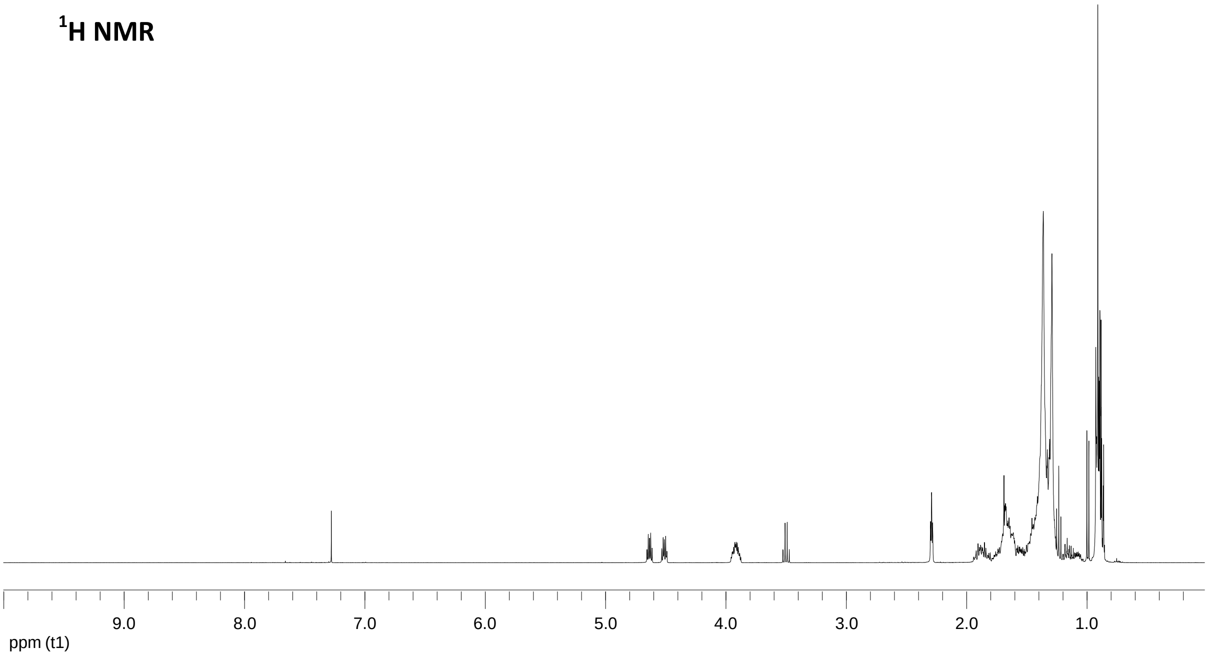

^{13}C NMR

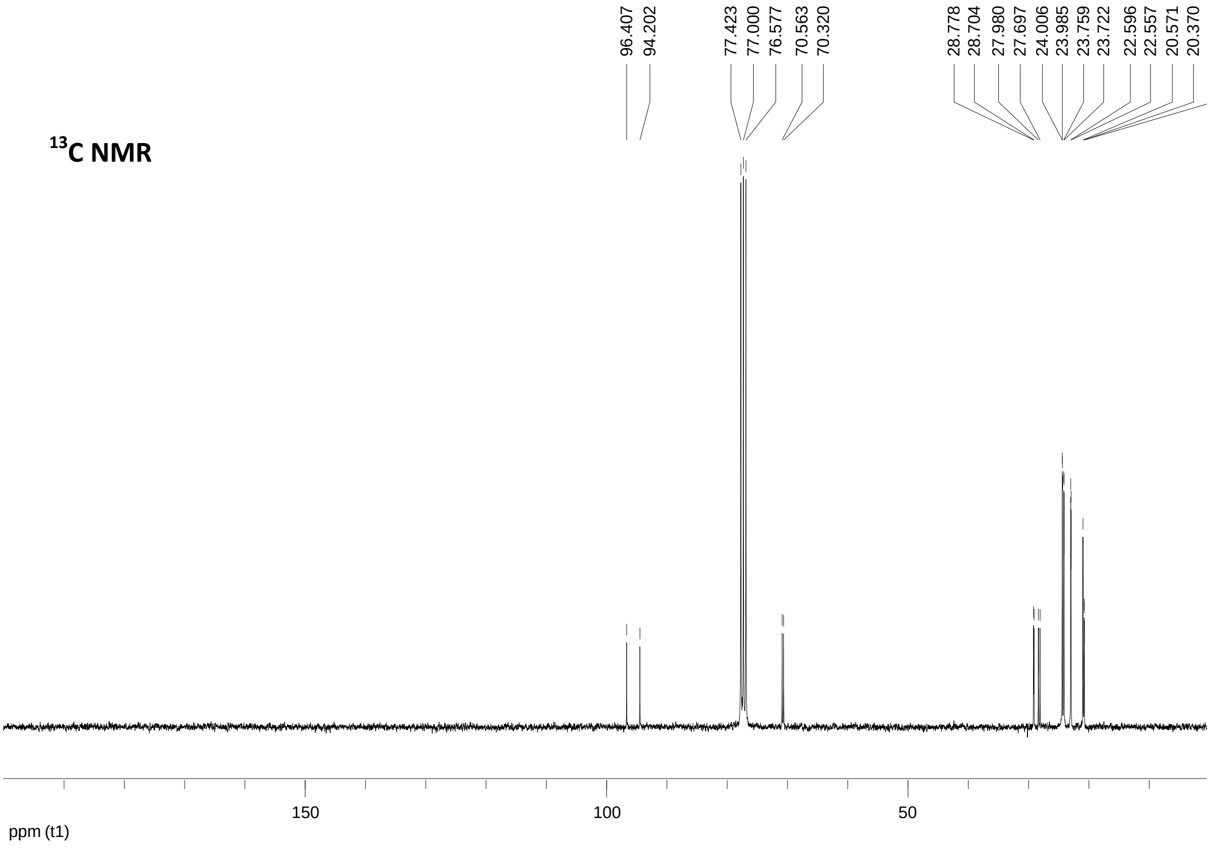

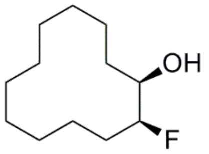

cis-**7b**

^{1}H NMR

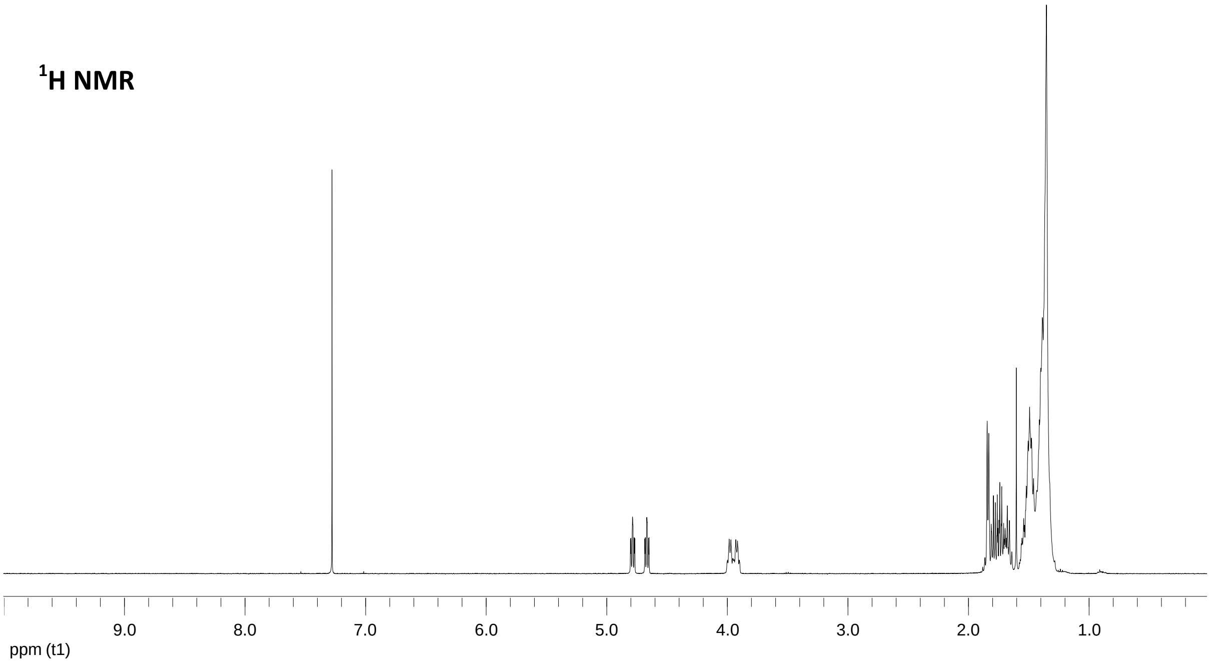

^{13}C NMR

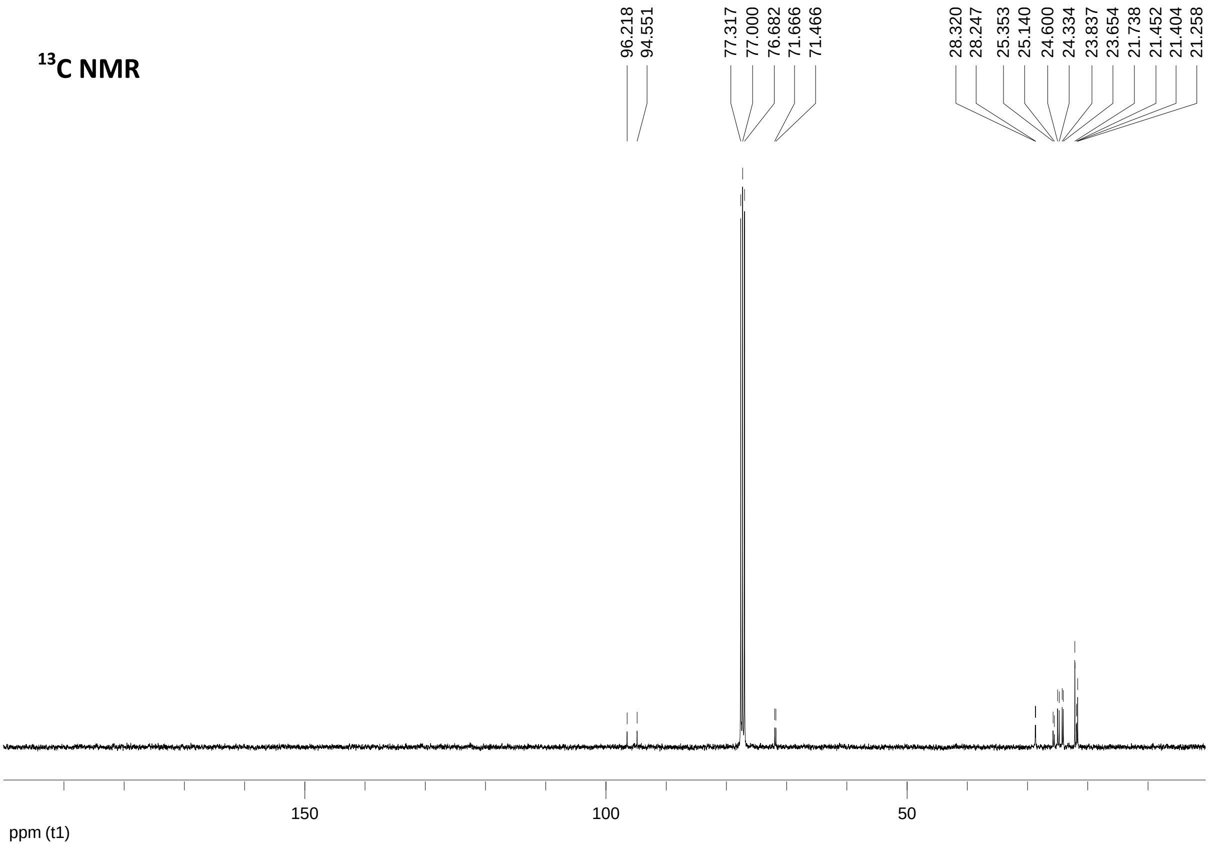

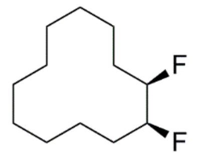

Erythro-**5a**

^{1}H NMR

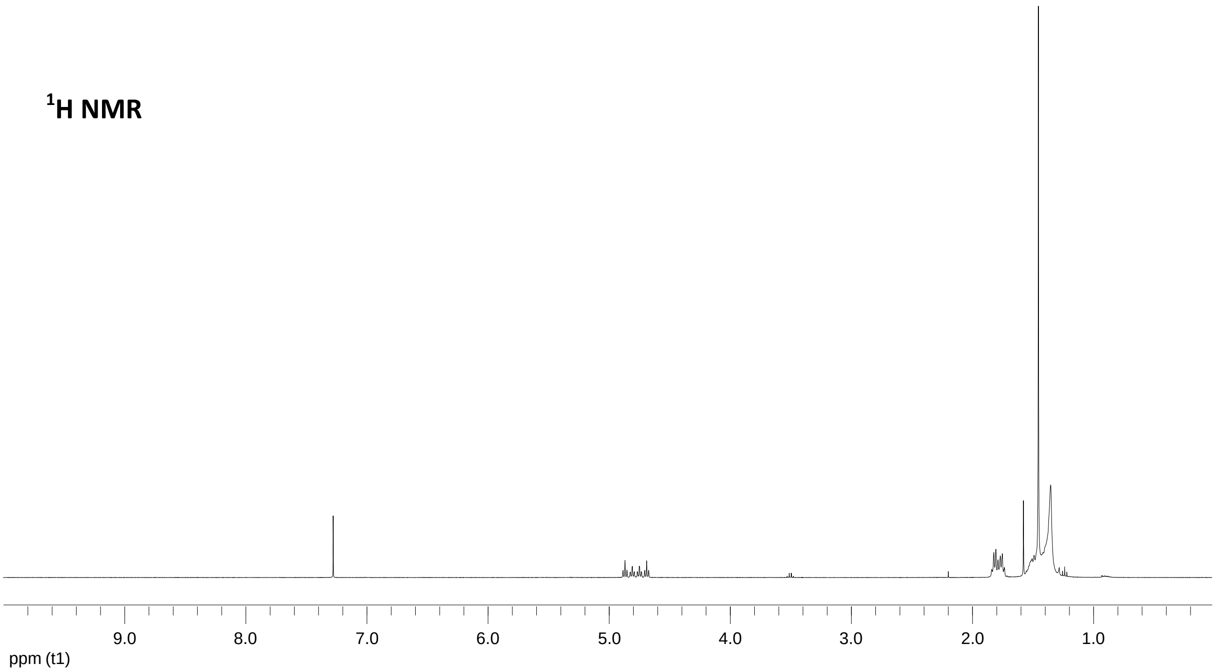

^{13}C NMR

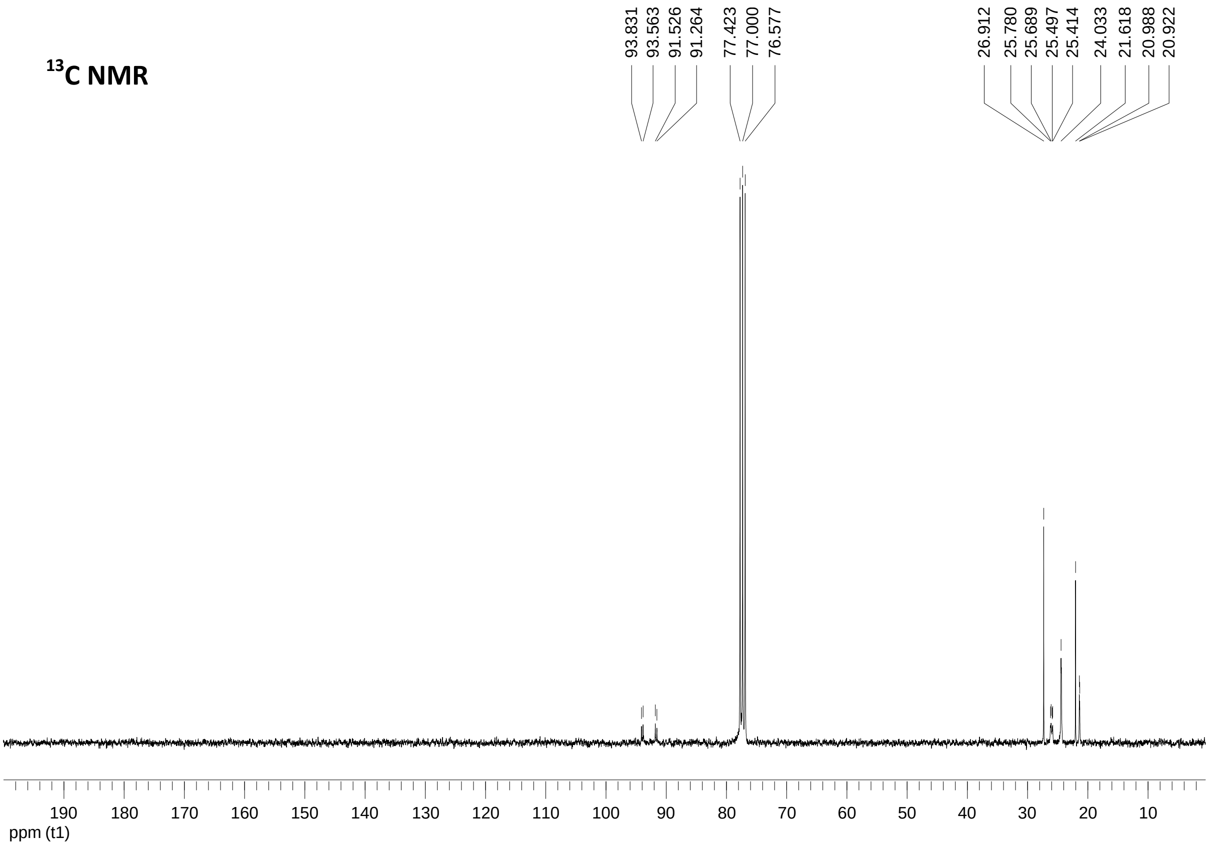

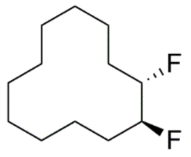

Threo-**5b**

^{1}H NMR

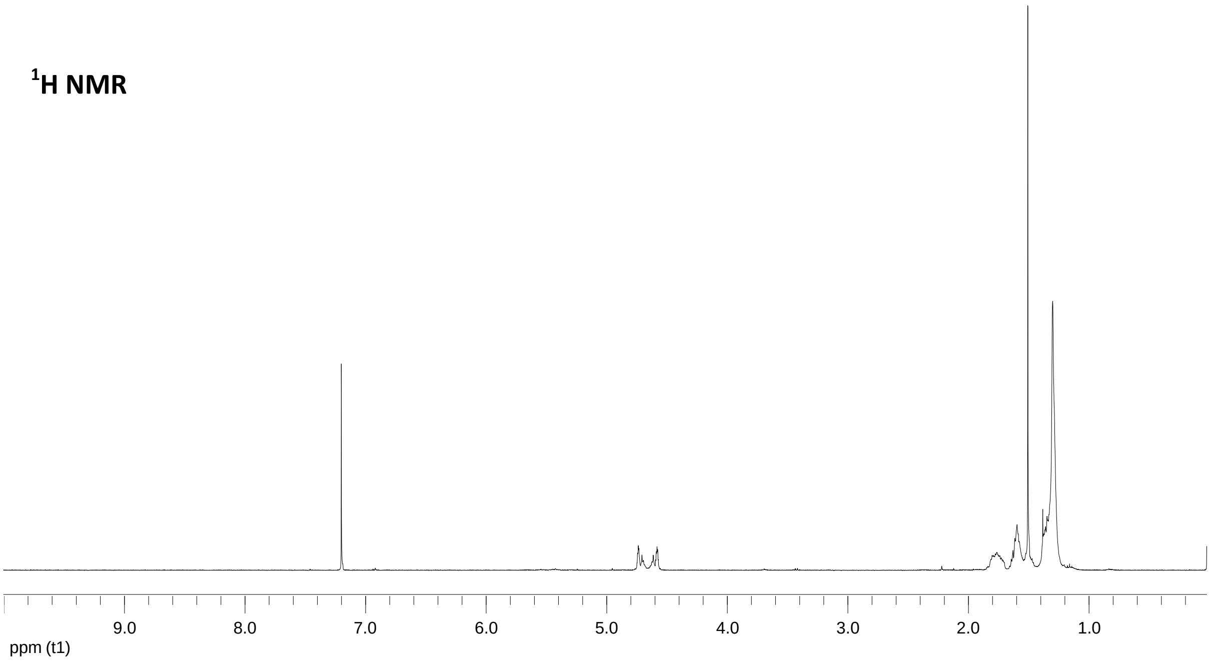

^{13}C NMR

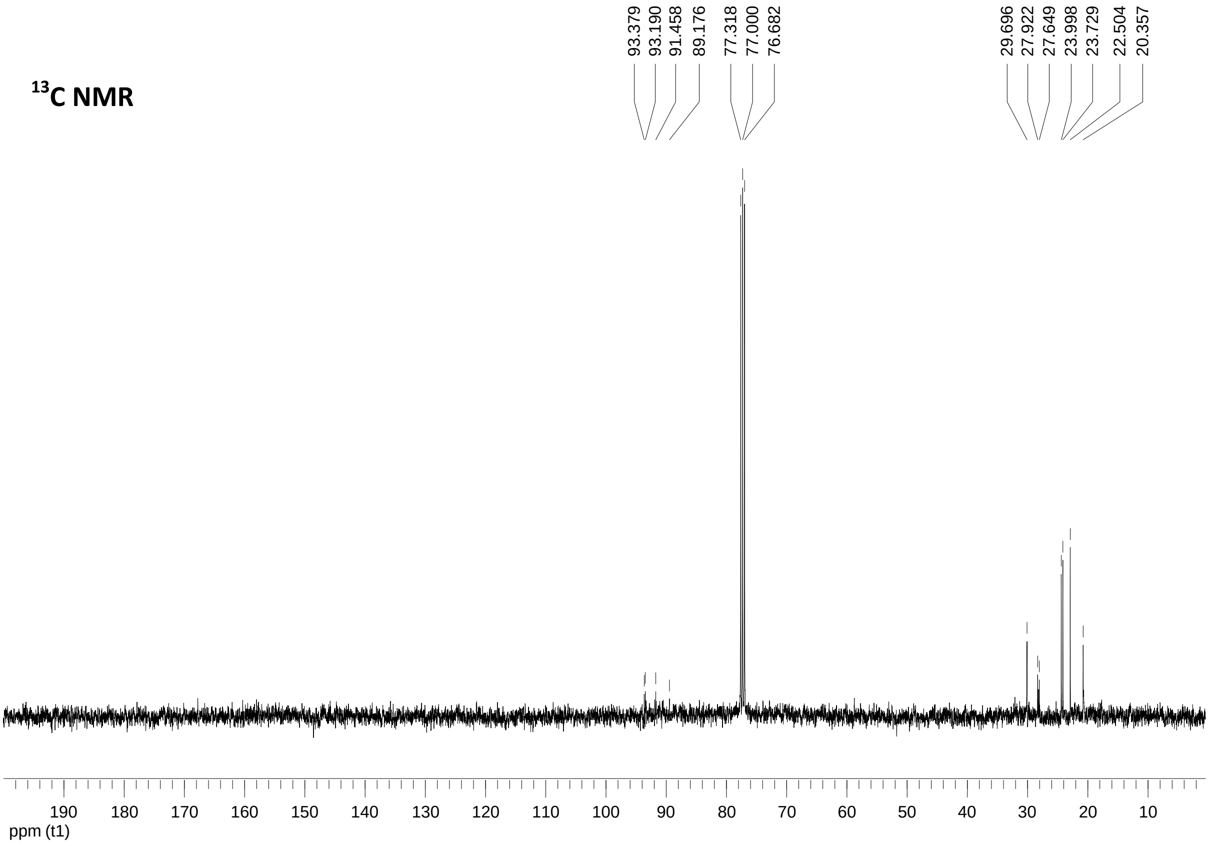

Section 2   Differential scanning calorimetry (DSC)

Measured on Netzsch DSC 204 Instrument with temperature range of −150 to 400 °C

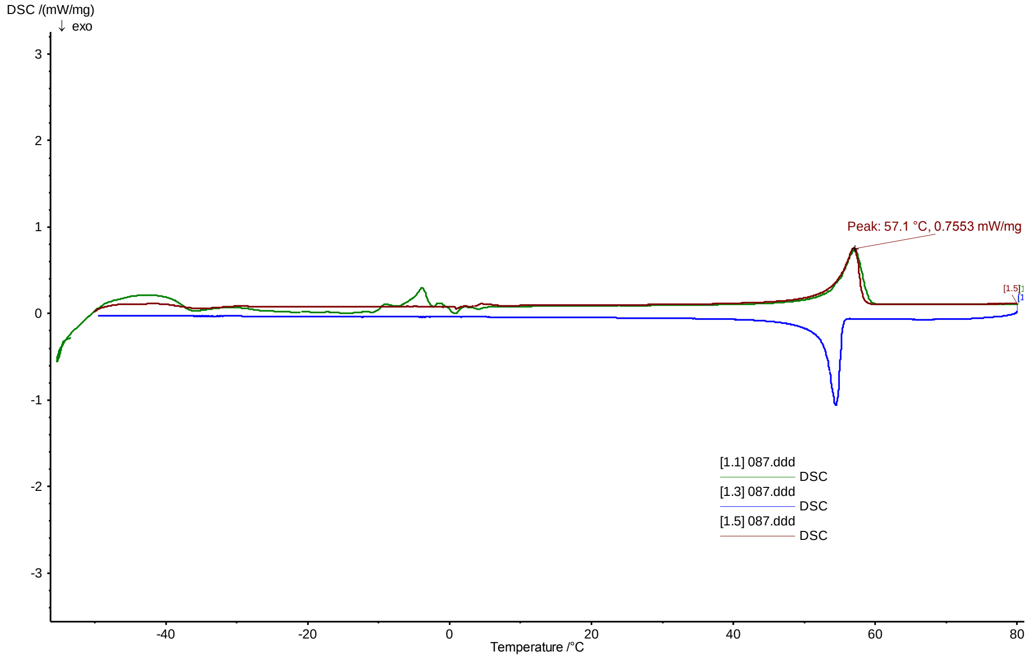

DSC of *erythro* cyclododecane 5a

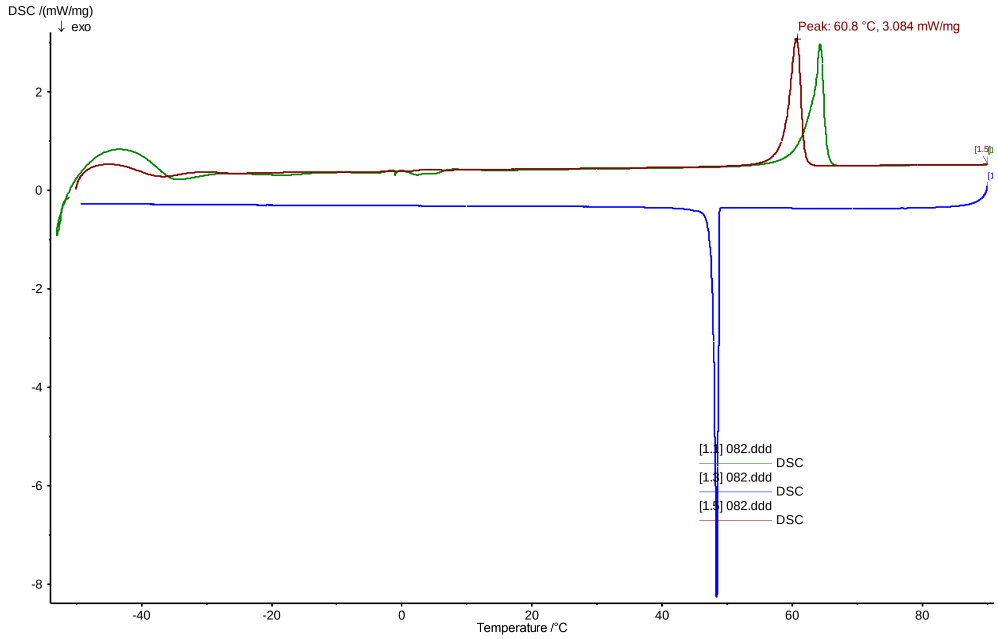

DSC of *threo*-cyclododecane 5b

Section 3 Variable-temperature NMR

Variable-temperature ^{19}F NMR experiments of *erythro*-fluorocyclododecane 5a.

	Chemical shifts			
Exp No	*T* [K]	*k* [Hz]	1/*T*	ln(*k*/*T*)
1	200.38	11.8	0.004991	−2.83212
2	200.38	20.0	0.004991	−2.30448
3	204.05	31.3	0.004901	−1.87475
4	209.06	64.9	0.004783	−1.16977
5	212.85	96.8	0.004698	−0.78828
6	220.30	201.8	0.004539	−0.08776
7	224.59	296.8	0.004453	0.278782
8	232.11	561.7	0.004308	0.883751
9	236.16	837.7	0.004234	1.26618
10	241.79	1208.5	0.004136	1.609024

Fitting experimental data to the Eyring equation provides activation parameters of the ring interconversion process $\Delta G^{\#}_{298}$ = 44.1 ± 6.1 kJ·mol^{-1} (10.5 ± 1.5 kcal·mol^{-1}), $\Delta G^{\#}_{203}$ = 48.1 kJ·mol^{-1} (11.4 kcal·mol^{-1}), $\Delta H^{\#}$ = 56.6 ± 2.7 kJ·mol^{-1} (13.5 ± 0.6 kcal·mol^{-1}), and $\Delta S^{\#}$ = 42.0 ± 11.3 J·K^{-1}·mol^{-1} (10.0 ± 2.7 kcal·mol^{-1}).

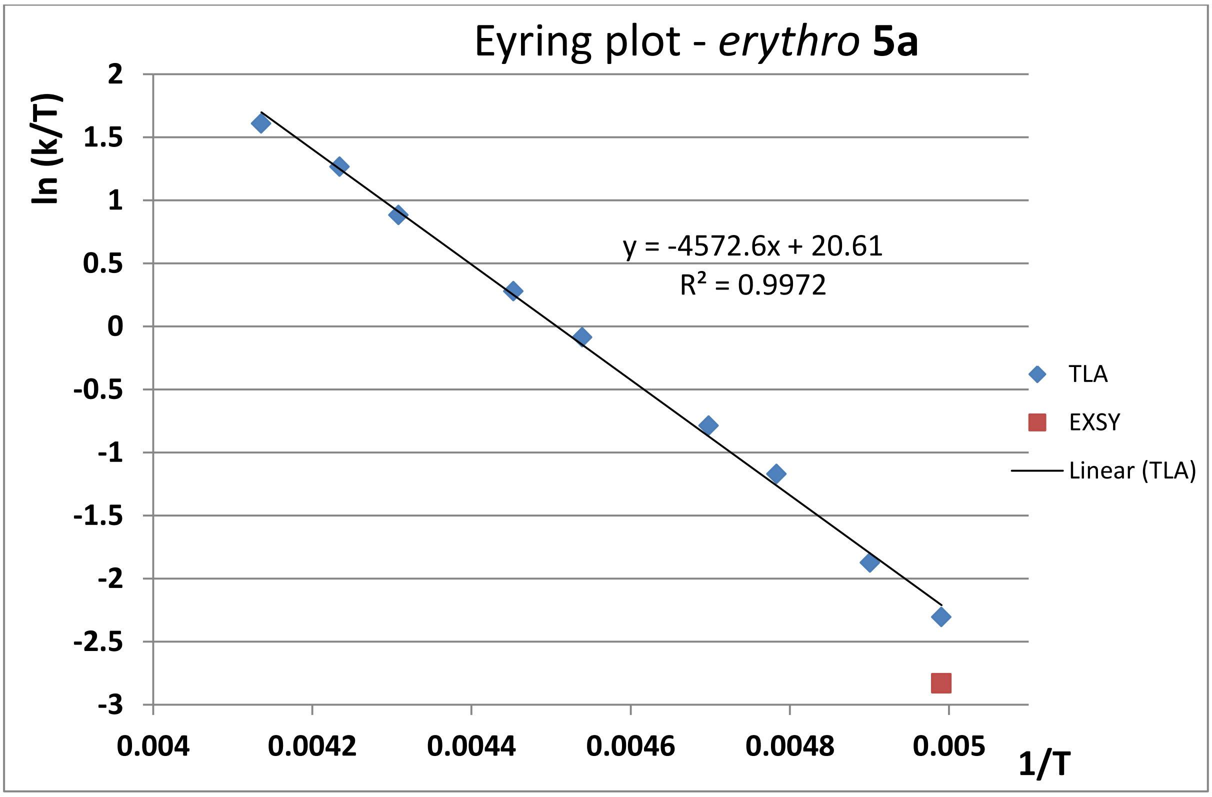

Variable-temperature ^{19}F NMR experiments of *threo*-difluorocyclododecane 5b

The ^{19}F spectra in CD_2Cl_2 were recorded by using a Bruker AVANCE 500 MHz spectrometer equipped with 5 mm QNP-probe across the temperature range 193–219 K. The accurate temperatures for particular experiments were determined by using 4% MeOH in methanol-d_4 sample. Complete lineshape analysis was carried out by using a Bruker Topspin D-NMR module.

	Chemical shifts					
Exp No	A	B	*T* [K]	*k* [Hz]	1/*T*	ln(*k*/*T*)
1	−192.6185	−194.1307	184.31	481.5	0.005426	0.960266
2	−192.5417	−194.1795	191.31	774.7	0.005227	1.398568
3	−192.4557	−194.2260	196.26	1219.2	0.005095	1.826534
4	−192.4435	−194.3793	200.87	2224.2	0.004978	2.404504
5	−192.5098	−194.4943	206.06	3400.6	0.004853	2.80354

Fitting experimental data to the Eyring equation provides activation parameters of the ring interconversion process $\Delta G^{\#}_{298}$ = 39.8 ± 4.8 kJ·mol^{-1} (9.47 ± 1.1 kcal·mol^{-1}), $\Delta G^{\#}_{203}$ = 35.9 kJ·mol^{-1} (8.55 kcal·mol^{-1}), $\Delta H^{\#}$ = 27.6 ± 1.2 kJ·mol^{-1} (6.57 ± 0.3 kcal·mol^{-1}), and $\Delta S^{\#}$ = -40.9 ± 12.0 J·K^{-1}·mol^{-1} (-9.73 ± 2.9 kcal·mol^{-1}).

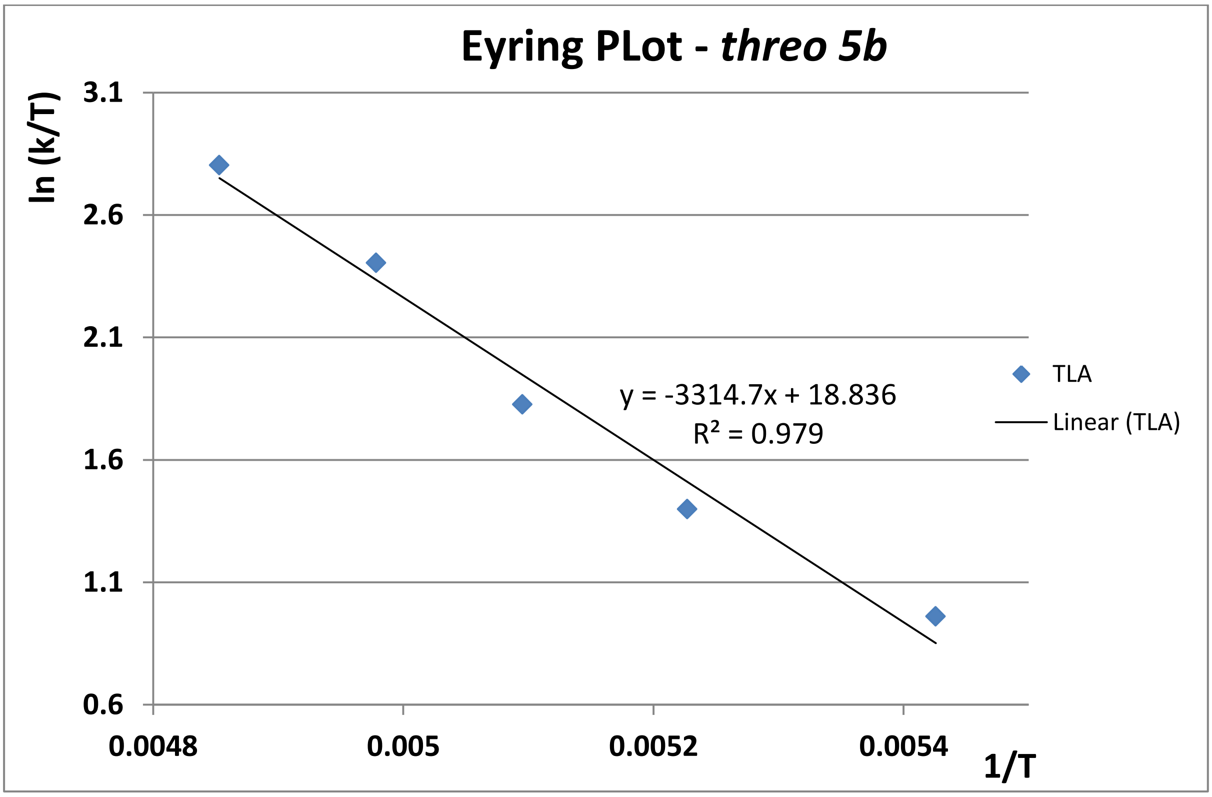

Section 4 Computational studies for each conformer of 1,2-difluorocyclododecane

Geometry 1	cC12-corner-aa B3LYP/6-311+G(2d,p)	cC12-corner-ae B3LYP/6-311+G(2d,p)	cC12-corner-ea B3LYP/6-311+G(2d,p)	cC12-corner-ee B3LYP/6-311+G(2d,p)	cC12-edge-aa B3LYP/6-311+G(2d,p)	cC12-edge-ee B3LYP/6-311+G(2d,p)	cC12-edge-ae B3LYP/6-311+G(2d,p)
E (a.u.)	-670.42504150	-670.43041090	-670.42599480	-670.42881540	-670.42055320	-670.42798090	-670.42658030
E (kcal/mol)	-420691.71354125	-420695.08283975	-420692.31173700	-420694.08166350	-420688.89713300	-420693.55801475	-420692.67913825
ZPE (kcal/mol)	204.76313000	204.46113000	204.76329000	204.46151000	204.74204000	204.70837000	204.63087000
E+ZPE (kcal/mol)	-420486.95041125	-420490.62170975	-420487.54844700	-420489.62015350	-420484.15509300	-420488.84964475	-420488.04826825
**rel E (kcal/mol)**	**3.67**	**0.00**	**3.07**	**1.00**	**6.47**	**1.77**	**2.57**

Geometry 1	cC12-corner-aa MP2/6-311+G(2d,p)	cC12-corner-ae MP2/6-311+G(2d,p)	cC12-corner-ea MP2/6-311+G(2d,p)	cC12-corner-ee MP2/6-311+G(2d,p)	cC12-edge-aa MP2/6-311+G(2d,p)	cC12-edge-ee MP2/6-311+G(2d,p)	cC12-corner-ae MP2/6-311+G(2d,p)
E (a.u.)	-668.62448390	-668.62992400	-668.62565740	-668.62858320	-668.61977200	-668.62786340	-668.62571840
E (kcal/mol)	-419561.86364725	-419565.27731000	-419562.60001850	-419564.43595800	-419558.90693000	-419563.98428350	-419562.63829600
ZPE (kcal/mol)	204.76313000	204.46113000	204.76329000	204.46151000	204.74204000	204.70837000	204.63087000
E+ZPE (kcal/mol)	-419357.10051725	-419360.81618000	-419357.83672850	-419359.97444800	-419354.16489000	-419359.27591350	-419358.00742600
**rel E (kcal/mol)**	**3.72**	**0.00**	**2.98**	**0.84**	**6.65**	**1.54**	**2.81**

Geometry 1	cC12-corner-aa M06-2X/6-311+G(2d,p)	cC12-corner-ae M06-2X/6-311+G(2d,p)	cC12-corner-ea M06-2X/6-311+G(2d,p)	cC12-corner-ee M06-2X/6-311+G(2d,p)	cC12-edge-aa M06-2X/6-311+G(2d,p)	cC12-edge-ee M06-2X/6-311+G(2d,p)	cC12-edge-ae M06-2X/6-311+G(2d,p)
E (a.u.)	-670.130765	-670.1349674	-670.1319447	-670.1342023	-670.1275112	-670.1333177	-670.132035
E (kcal/mol)	-420507.055	-420509.692	-420507.7953	-420509.2119	-420505.0133	-420508.6569	-420507.852
ZPE (kcal/mol)	206.97359	206.67512	207.02632	206.90772	207.18351	206.64104	207.00078
E+ZPE (kcal/mol)	-420300.0814	-420303.0169	-420300.769	-420302.3042	-420297.8298	-420302.0158	-420300.8512
**rel E (kcal/mol)**	**2.94**	**0.00**	**2.25**	**0.71**	**5.19**	**1.00**	**2.17**
